# Barriers and facilitators of cross-sectoral data linkage to inform healthy public policy and practice: lessons from three case study projects in Scotland.

**DOI:** 10.23889/ijpds.v7i3.2062

**Published:** 2022-08-25

**Authors:** Kristina Cimova, David Henderson, Mirjam Allik, Denise Brown, Petra Meier, Charlie Mayor, Nick Watson, Peter Craig, Emily Tweed

**Affiliations:** 1 MRC/CSO Social and Public Health Sciences Unit, University of Glasgow; 2 Usher Institute, University of Edinburgh; 3 School of Social and Political Sciences, University of Glasgow

## Objectives

We sought to describe barriers and facilitators faced by three research projects aiming to link routinely-collected data across various sectors, to produce evidence to inform healthy public policy. We conducted these case studies as a part of a wider research project on cross-sectoral sharing and linkage of secondary data t.

## Approach

We selected the case studies to cover a range of target populations and datasets. The chosen projects investigated (1) the health of care-experienced children; (2) the intersection of homelessness, justice involvement, drug use, and severe mental illness; (3) multi-morbidity among adults receiving social care. Information about timelines and governance processes was collected from lead investigators, including specific barriers and facilitators encountered, using a standardised pro forma and follow-up interviews. Thematic analysis was carried out by the research team, informed by themes identified in a parallel scoping review of existing literature on evidence use for healthy public policy and practice.

## Results

Each project involved between 6 and 11 agencies, with co-ordination across multiple institutions and geographies proving challenging. Due to challenges encountered, all projects had to amend their original geographical or demographic scope. Forty-four barriers and facilitators to sharing and linkage of cross-sectoral routinely-collected data for public health research were identified. These included but were not limited to: integration of current data in an ever-changing linkage landscape; the need for timely feedback in undertaking the study; standardisation of information governance processes; highlighting the resourcing and funding issues for data linkage projects; the need for data controllers to recognise the value of such projects; and issues relating to staff turnover and workload pressures.

## Conclusion

The interconnected nature of barriers and facilitators identified by the case studies suggests the importance of a whole-systems approach to cross-sectoral linkage. While literature offers relatively few case studies of cross-sectoral linkage for health research, the value of their insight into the linkage landscape derived from real-life experience is substantial.

